# Supplementary material for: Identification of photoperiod-induced specific miRNAs in the adrenal glands of Sunite sheep (Ovis aries)
Source: Front Vet Sci. 2022 Jul 22;9:888207. doi: 10.3389/fvets.2022.888207 (PMC9354845; doi:10.3389/fvets.2022.888207)
Supplement: Supplementary file 1 [file Data_Sheet_1.ZIP › Supplementary file/miRDeep2 file.zip!/miRDeep2 file/Know_Structure/LP42A1/oar-mir-374b.pdf]

miRBase precursor	: oar-mir-374b
Total read count	: 31578
oar-miR-374b read count	: 30914
remaining reads	: 664

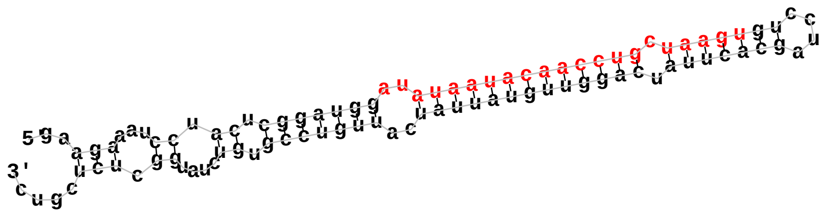

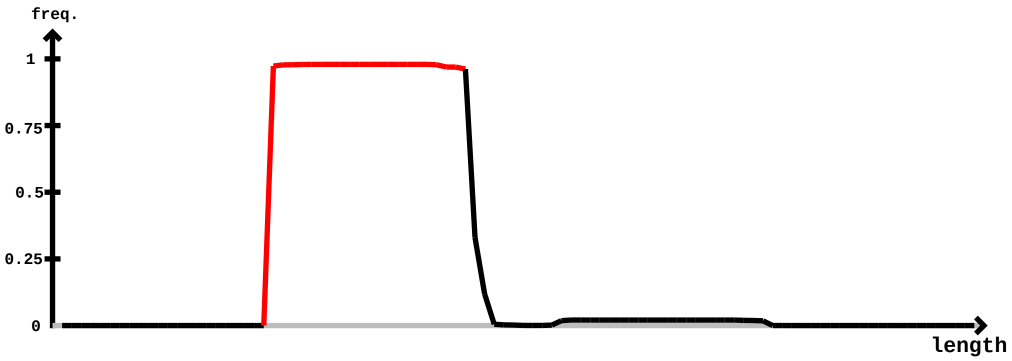

oar-miR-374b

5'- gaagaaauccuacucggauggauauaauacaaccugcuaaguguccuagcacuuaucagguuguauuaucauuguccgugucuauggcucucguc -3'	exp		
((((..((...((.(((((((..((((((((((((.(((((((....))))))).))))))))))))..))))))).))..))..)).))...	reads	mm	sample
.....................auauaauacaaccugcu...	41	0	seq
.....................Cuauaauacaaccugcu...	4	1	seq
.....................auauaauaAaaccugcu...	1	1	seq
.....................auauaauacaaccugcA...	3	1	seq
.....................auauaauacaaccugcua..	265	0	seq
.....................auauaauacaaccugcuaa...	12	0	seq
.....................auauaauacaaccugcuaag..	227	0	seq
.....................auauaauacaaccugcuaagu...	19947	0	seq
.....................auauaauacaaccugcuaagug..	6670	0	seq
.....................auauaauacaaccugcuaagugu...	3483	0	seq
.....................auauaauacaaccugcuaaguguc..	55	0	seq
.....................auauaauacaaccugcuaagugucc...	4	0	seq
.....................auauaauacaaccugcuaaguguccu..	8	0	seq
.....................auauaauacaaccugcuaaguguccuagc...	3	0	seq
.....................auauaauacaaccugcuaaguguccuagcacu..	3	0	seq
.....................auauaauacaaccugcuaaguguccuagcacuuauc......................................	2	0	seq
......................uauaauacaaccugcuaagu...	14	0	seq
......................uauaauacaaccugcuaagug..	4	0	seq
......................uauaauacaaccugcuaagugu...	92	0	seq
......................uauaauacaaccugcuaaguguc..	16	0	seq
......................uauaauacaaccugcuaagugucc...	5	0	seq
......................uauaauacaaccugcuaaguguccu..	6	0	seq
......................uauaauacaaccugcuaaguguccuagcacuuauc......................................	1	0	seq
.......................auaauacaaccugcuaagu...	2	0	seq
.......................auaauacaaccugcuaagug..	3	0	seq
.......................auaauacaaccugcuaagugu...	4	0	seq
.......................auaauacaaccugcuaaguguc..	3	0	seq
.......................auaauacaaccugcuaagugucc...	2	0	seq
.......................auaauacaaccugcuaaguguccu..	1	0	seq
........................uaauacaaccugcuaaguguc..	1	0	seq
........................uaauacaaccugcuaagugucc...	6	0	seq
........................uaauacaaccugcuaaguguccu..	23	0	seq
........................uaauacaaccugcuaaguguccua...	1	0	seq
........................uaauacaaccugcuaaguguccuagc...	1	0	seq
.........................aauacaaccugcuaagugucc...	1	0	seq

oar-miR-374b

```
gaagaaauccuacucggauggauauaauacaaccugcuaaguguccuagcacuuaucagguuguauuaucauuguccgugucuauggcucucguc
.........................aauacaaccugcuaaguguccu................................................   9     0   seq
..........................auacaaccugcuaaguguccu................................................   2     0   seq
..........................auacaaccugcuaaguguccua...............................................   1     0   seq
..........................auacaaccugcuaaguguccuag..............................................   1     0   seq
..........................auacaaccugcuaaguguccuagcacuu.........................................   1     0   seq
...........................uacaaccugcuaaguguccuag..............................................   1     0   seq
.................................................cacuuaucagguuguauuauc.........................   3     0   seq
.................................................cacuuaucagguuguauuauca........................   2     0   seq
..................................................Ccuuaucagguuguauuauc.........................   3     1   seq
..................................................acuuaucagguuguauuauA.........................   2     1   seq
..................................................acuuaucagguuguauuauc.........................   16    0   seq
..................................................acuuaucagguuguauuauca........................   4     0   seq
..................................................acuuaucagguuguauuaucau.......................   17    0   seq
..................................................acuuaucagguuguauuaucauu......................   1     0   seq
...................................................cuuaucagguuguauuauc.........................   6     0   seq
...................................................cuuaucagguuguauuauca........................   8     0   seq
...................................................cuuaucagguuguauuauAa........................   1     1   seq
...................................................cuuaucagguuguauuaucau.......................   24    0   seq
...................................................cuuaucagguuguauuaucauu......................   486   0   seq
....................................................uuaucagguuguauuauc.........................   2     0   seq
....................................................uuaucagguuguauuauca........................   2     0   seq
....................................................uuaucagguuguauuaucau.......................   3     0   seq
....................................................uuaucagguuguauuaucauu......................   65    0   seq
....................................................uuaucagguuguauuaucauugu....................   2     0   seq
.....................................................uaucagguuguauuaucauu......................   1     0   seq
.....................................................uaucagguuguauuaucauugu....................   1     0   seq
```
